# Personality and Sleep Health: Do Lifestyle Habits Play a Role?

**DOI:** 10.1093/geroni/igaa057.1383

**Published:** 2020-12-16

**Authors:** Karley Deason, Christina Mu, Soomi Lee

**Affiliations:** University of South Florida, Tampa, Florida, United States

## Abstract

The health behavior model proposes that healthy/unhealthy behaviors may play a role in the relationship between personality and health. Previous research shows that personality traits are linked to sleep, however, few studies have considered the moderating role of unhealthy behaviors in the personality—sleep relationship. The current study investigated the associations between specific personality traits and sleep and whether the associations were moderated by unhealthy behaviors. Participants were 61 oncology nurses (Mage=35.39, SDage=11.73). They responded to a background survey that assessed the big five personality traits and engagement in unhealthy behaviors (i.e., exercise, smoking, fast food and alcohol consumption). For two weeks, ecological momentary assessments captured daily variability in sleep (i.e., quality, sufficiency, onset latency, insomnia, duration). A series of multilevel models was used. After controlling for sociodemographics and work shift, higher conscientiousness was associated with greater sleep sufficiency (B=0.31, p<.05) and lower odds of having insomnia symptoms (OR=0.24, p<.05). Moreover, higher agreeableness was associated with longer sleep duration (B= 0.51, p<.05) and lower odds of insomnia symptoms (OR=0.29, p<.05). Other personality domains were not associated with sleep, however, extraversion interacted with unhealthy behaviors to be associated with sleep. Those who were more extraverted reported lower odds of insomnia and better sleep sufficiency; these associations were significant only for those with less unhealthy behaviors. Findings suggest that conscientiousness and agreeableness were associated with sleep health. The interaction between extraversion and unhealthy behaviors suggests that reducing unhealthy behaviors may be beneficial to improving sleep in individuals with certain personality traits.

